# Bony Morphological Features of the Proximal Femur in Patients With Developmental Dysplasia of the Hip

**DOI:** 10.7759/cureus.81410

**Published:** 2025-03-29

**Authors:** Hideki Shozen, Takeshi Shoji, Shinichi Ueki, Hiroki Kaneta, Nobuo Adachi

**Affiliations:** 1 Orthopedic Surgery, Hiroshima University Hospital, Hiroshima, JPN

**Keywords:** bone morphology, ct based image analysis, developmental dysplasia of the hip ( ddh ), lesser trochanter, proximal femur morphology

## Abstract

Purpose

Developmental dysplasia of the hip (DDH) is a precursor of secondary osteoarthritis, with borderline DDH (BDDH) often overlooked. This study explores three-dimensional (3D) proximal femoral morphology in DDH and BDDH and examines their distinctions from normal hips (NH), emphasizing new correlations between femoral parameters and hip instability.

Methods

We retrospectively analyzed 130 hips from 120 patients categorized as DDH (lateral center-edge angle (LCEA) < 20°), BDDH (20°-25°), or NH (>25°). Detailed 3D analyses using advanced CT imaging measured unique femoral parameters such as femoral neck anteversion (FAV), lesser trochanter version (LTV), and femoral head morphology. Statistical methods assessed differences among groups and identified novel correlations.

Results

The study confirmed that DDH is associated with increased FAV and decreased LTV, alongside reduced femoral head-lesser trochanter distance (FH-LT) and an elliptical femoral head. BDDH displayed intermediate morphological changes, highlighting its biomechanical significance. Correlations revealed new insights into the relationship between hip morphology and clinical symptoms.

Discussion

External rotational abnormalities, such as increased FAV and decreased LTV, may predispose DDH patients to ischiofemoral impingement, psoas bursitis, and joint degeneration. The shortened FH-LT and elliptical femoral head further contribute to instability and early osteoarthritis. These findings underscore the role of 3D proximal femoral morphology in DDH biomechanics and clinical symptoms.

Conclusion

This study extends previous research by identifying previously unreported associations between proximal femoral abnormalities and hip biomechanics in DDH and BDDH. These findings inform diagnostic and therapeutic strategies for early intervention.

## Introduction

Developmental dysplasia of the hip (DDH) is a common cause of secondary hip osteoarthritis [1]. The disease spectrum ranges from acetabular dysplasia or subluxation to dislocation [2]. Many studies reported pelvic morphological abnormalities in patients with DDH. Fujii et al. reported a short distance between the bilateral anterior superior iliac spine (ASIS) and low-angle iliac wing on the axial and sagittal planes using computed tomography (CT), indicating internal rotation of the iliac wing in DDH [3]. Sako et al. reported a greater distance between the centers of the femoral head in patients with DDH than in normal hips [4]. When the ilium is internally rotated, the acetabular position should be internally rotated, and the distance between the centers of the femoral head should be equally shortened. Sako et al. reported that patients with DDH had an internal rotation of the ilium, ischiopubic bone, and acetabulum; however, the internal rotation decreased near the acetabulum, causing the lateral acetabular shift, resulting in a greater distance between the centers of the femoral head [4]. Furthermore, patients with DDH have shallow hip sockets, which lead to hip instability. Laborie et al. reported that patients with DDH had a low acetabular head index, which was associated with clinical hip instability [5]. Other examinations showed that deficiency of the bony acetabulum caused hip instability and acetabular rim overload with subsequent damage to the labrum and articular cartilage [6]. DDH tends to cause femoroacetabular impingement (FAI). Crawford et al. reported that radiographs of the hip joints that caused FAI had an elliptical femoral head or pistol grip deformity, and these radiographs frequently showed DDH [7].

Several reports have shown that there are not only pelvic morphological abnormalities but also proximal femoral abnormalities, such as an anteverted femoral neck and a deformed femoral head in patients with DDH. These features were reported to be associated with instability and impingement, although little information is available in terms of the whole morphology of the proximal femur in patients with DDH, which may affect hip instability, impingement, and clinical symptoms [8,9]. Despite the existence of proximal femoral morphology studies, no studies have evaluated the proximal femoral morphology in patients with borderline DDH (BDDH) [10,11]. This study aimed to evaluate the three-dimensional (3D) proximal femoral bony morphology in DDH and assess the differences in the proximal femoral morphology between normal hip (NH), DDH, and BDDH.

## Materials and methods

A retrospective analysis was performed on all patients who underwent medical examinations at Hiroshima University Hospital between 2011 and 2023. CT images were obtained as a standard protocol for surgical support using a helical CT scanner (Aquilion ONE, Toshiba Medical Healthcare, Tochigi, Japan) and set as axial images, with slice thicknesses of 2 mm from the iliac brim to the knee joint. We had ethical approval and informed consent. The CT images of the hip joint, obtained during pre-operative examinations, were reviewed from 120 patients (130 hips) with DDH (lateral center-edge angle: LCEA < 20°), BDDH (LCEA 20-25°), and NH (LCEA > 25°) (Table 1). The LCEA was measured in the coronal plane and passed through the center of the femoral head in the anterior pelvic plane (APP). The DDH and BDDH groups included patients with pre- and early-stage osteoarthritis (according to the Japanese Orthopaedic Association classification) who had no medical history of hip joint dislocation. Patients in the NH group had no hip diseases, such as trauma or osteonecrosis of the femoral head, or had undergone total hip arthroplasty (THA). A CT-based simulation software (LEXI® Co., Ltd., Tokyo, Japan) was used to create virtual 3D bone models and evaluate the femoral bone morphology [12].

**Table 1:** Patient demographics DDH: developmental dysplasia of the hip; BDDH: borderline DDH; NH: normal hip; BMI: body mass index

Demographic	DDH (n=50)	BDDH (n=30)	NH (n=50)
Age mean	49.0	49.1	51.0
Male:Female	5 hips:45 hips	8 hips:22 hips	31 hips:19 hips
Right:Left	29:21	16:14	15:35
Height mean (cm)	157	159	163
Weight mean (kg)	55.8	61.7	63.1
BMI mean	22.5	24.3	23.7

The following parameters were measured using the software: pelvic parameters, femur parameters, the neck-shaft angle (NSA), modified NSA (mNSA), femoral neck anteversion (FAV), lesser trochanter version (LTV), length of the femoral head center to the lesser trochanter (FH-LT), alpha angle (αA), canal flare index (CFI), femoral head diameter (FHD), flattened rate of the femoral head (FR of FH), femoral offset (FO), and ischiofemoral space (IFS).

**Figure 1:**
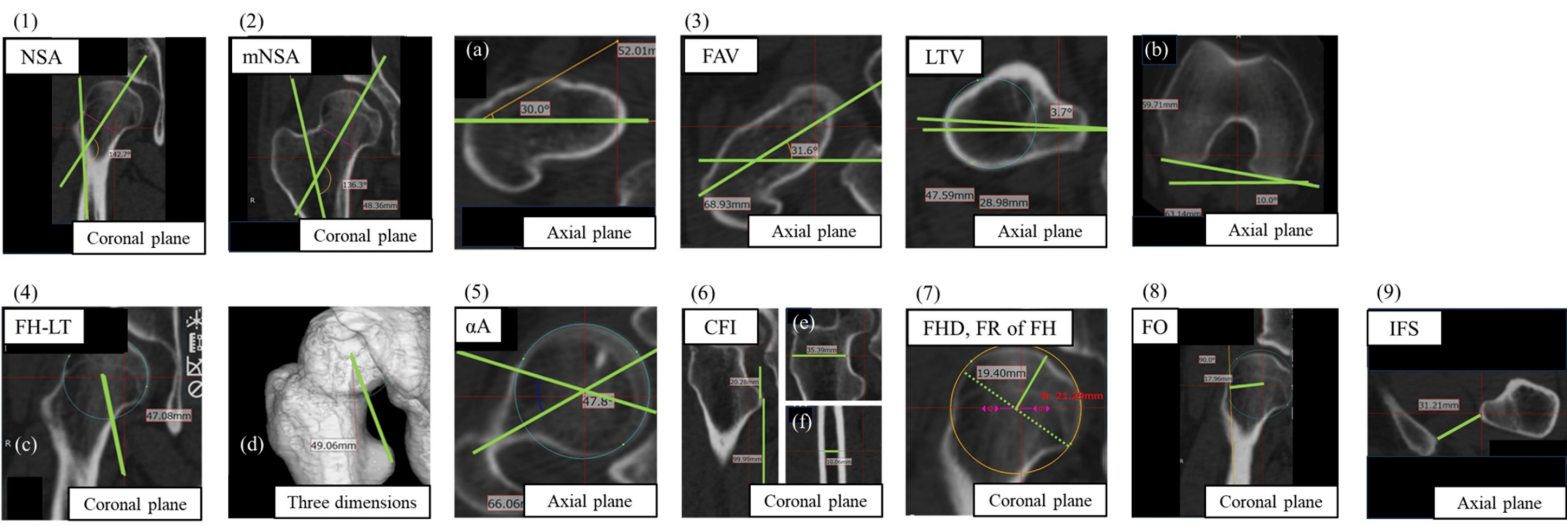
Definition of the measurements 1: neck-shaft angle (NSA) is the angle between the femoral neck axis and the central axis of the femoral medullary canal; 2: modified NSA (mNSA) is NSA, which was standardized by femoral anteversion 0° (a); 3: femoral neck anteversion (FAV) is the angle between a midline through the femoral neck and the femoral condylar axis, lesser trochanter version (LTV) is the angle between the line connecting the femoral bone shaft through the tip of the lesser trochanter and the femoral condylar axis, FAV and LTV are modified by a tangent line between the femoral posterior condyles (b); 4: length from the femoral head center to the lesser trochanter (FH-LT) is the distance from the center of the femoral head to the tip of the lesser trochanter, which is measured by the coronal plane (c) and three dimensions (d); 5: alpha angle (αA) is the angle between the central axis of the femoral medullary canal and the line connecting the center of the femoral head through the first point where any bone deviated from the femoral head; 6: canal flare index (CFI) is the ratio of the medullary cavity widths at 2 cm above the tip of the lesser trochanter (e) to the medullary cavity widths at 10 cm below the tip of the lesser trochanter (f); 7: femoral head diameter (FHD) is the diameter at center of the femoral head (dashed line), flattened rate of the femoral head (FR of FH) is the ratio of the minor axis (solid line) to the major axis (half of the dashed line); 8: femoral offset (FO) is the distance from the center of the femoral head to the femoral shaft; 9: ischiofemoral space (IFS) is the distance from the tip of the lesser trochanter to the ischium.

Figure 1 shows the measurement of these parameters. IFS were standardized by APP, NSA, FAV, LTV, FH-LT, αA, CFI, FHD, FR of FH, and FO were standardized by the International Society of Biomechanics (ISB) recommendations [13]. In addition, FAV and LTV were modified using a tangent line between the femoral posterior condyles.

Case selection

All cases were randomly selected from the hospital's archive, with strict adherence to the inclusion criteria. It is also important to note that while both unilateral and bilateral cases were included, some bilateral cases had different classifications (e.g., one side DDH and the other BDDH).

Measurement consistency

All measurements were performed by a single trained and experienced observer. While this may not represent the ideal approach, the observer’s expertise in the relevant imaging techniques ensured consistency and accuracy in the measurements.

Statistical analysis

All measurements were expressed as mean ± standard deviation. Differences in all parameters among the three groups were assessed using a one-way analysis of variance and the Tukey-Kramer multiple comparison test. Correlations between LCEA and parameters that were statistically significant in multiple comparison tests were analyzed using the Pearson’s product-moment correlation coefficient. Correlations between LTV and the two parameters (FAV and IFS) were also analyzed using Pearson’s product-moment correlation coefficient. Statistical significance was set at p < 0.05.

## Results

Table 2 summarizes the differences in femoral morphology among DDH, BDDH, and NH groups. The DDH group showed significantly lower LTV, FO, FH-LT2D, FH-LT3D, and FHD compared to NH, while NSA and FAV were significantly higher in the BDDH and NH groups. The FR of FH and IFS was highest in NH, and αA was lowest in BDDH. No significant differences were observed in mNSA and CFI.

**Table 2:** Results of measurements NSA: neck-shaft angle; mNSA: modified NSA; FAV: femoral anteversion; LTV: lesser trochanter version; FH-LT: distance from the center of the femoral head to the tip of the lesser trochanter; αA: alpha angle; CFI: canal flare index; FHD: femoral head diameter; FR of FH: flattened rate of the femoral head; FO: femoral offset; IFS: distance from the tip of the lesser trochanter to the ischium; DDH: developmental dysplasia of the hip; BBDH: borderline DDH; NH: normal hip

Parameters	DDH	BDDH	NH
NSA	137.6±5.6°	132.7±5.8°	131.2±5.4°
mNSA	134.3±4.7°	135.0±4.8°	136.0±2.0°
FAV	27.2±10.4°	19.5±8.1°	11.5±6.5°
LTV	9.3±7.6°	18.0±10.3°	23.0±7.5°
FH-LT2D	49.4±4.4 mm	51.3±6.1 mm	52.7±4.1 mm
FH-LT3D	52.2±4.0 mm	54.4±5.3 mm	55.4±3.9 mm
αA	51.6±12.3°	49.3±11.3°	56.9±10.2°
CFI	4.3±0.8	4.4±0.5	4.3±0.7
FHD	44.6±3.0 mm	44.7±2.8 mm	46.2±3.3 mm
FR of FH	92.5±5.6%	94.8±4.7%	98.3±2.7%
FO	26.4±5.8 mm	32.5±4.7 mm	35.9±5.0 mm
IFS	22.1±7.8 mm	25.6±9.2 mm	31.8±8.6 mm

**Figure 2:**
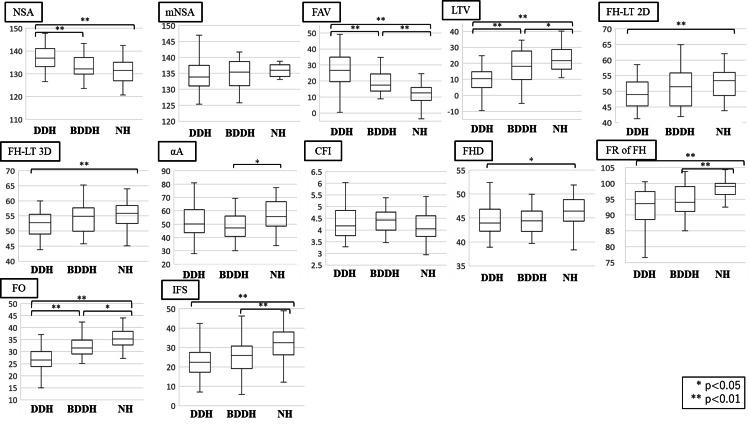
Results of measurements NSA: neck-shaft angle; mNSA: modified NSA; FAV: femoral anteversion; LTV: lesser trochanter version; FH-LT: distance from the center of femoral head to the tip of lesser trochanter; αA: alpha angle; CFI: canal flare index; FHD: femoral head diameter; FR of FH: flattened rate of femoral head; FO: femoral offset; IFS: ischiofemoral space The data in the box plots were represented as medians and interquartile ranges. Detailed statistical parameters, including means and standard deviations, are provided in Table 2. Statistical significance was set at p<0.05.

The LTV and FO in the DDH group were significantly lower than those in the BDDH and NH groups (p < 0.01). In contrast, the NSA and FAV were significantly higher in those in the BDDH and NH groups (p < 0.05). The FR of the FH and IFS in the NH group was significantly higher than that in the DDH and BDDH groups (p < 0.01). The FH-LT2D and FH-LT3D in the DDH group were significantly lower than those in the NH group (p < 0.01). The FHD in the DDH group was significantly lower than that in the NH group (p < 0.05). The αA in the BDDH group was significantly lower than that in the NH group (p < 0.01). The mNSA and CFI showed no significant differences among the three groups (Figure 2).

**Figure 3:**
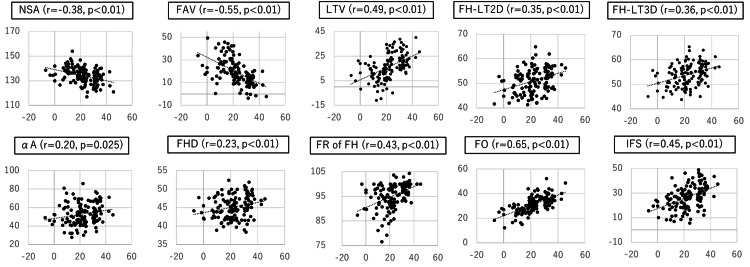
Correlation of LCEA with parameters LCEA: lateral center-edge angle; NSA: neck-shaft angle; FAV: femoral anteversion; LTV: lesser trochanter version; FH-LT: distance from the center of femoral head to the tip of lesser trochanter; αA: alpha angle; FHD: femoral head diameter; FR of FH: flattened rate of femoral head; FO: femoral offset; IFS: ischiofemoral space The scatter plots show the correlation between LCEA and each parameter. Data points represent individual measurements, and the dotted line indicates the trendline for the correlation. The correlation coefficient (r) and p-value are displayed in each panel. Statistical significance was set at p<0.05. Detailed statistical parameters, including means and standard deviations for each parameter, are presented in Table 2.

**Figure 4:**
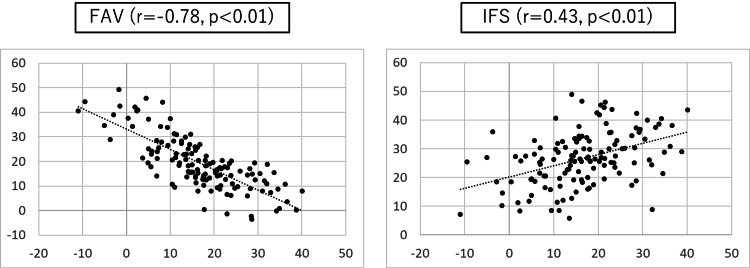
Correlation of LTV with FAV and IFS LTV: lesser trochanter version; FAV: femoral anteversion; IFS: distance from the tip of the lesser trochanter to the ischium The scatter plots show the correlation between LTV and each parameter. Data points represent individual measurements, and the dotted line indicates the trendline for the correlation. The correlation coefficient (r) and p-value are displayed in each panel. Statistical significance was set at p<0.05. Detailed statistical parameters, including means and standard deviations for each parameter, are presented in Table 2.

The correlation of the LCEA with the parameters that were statistically significant is shown in Figure 3. The LTV, FH-LT2D, FH-LT3D, αA, FHD, FR of FH, FO, and IFS had significant positive correlations with the LCEA, whereas the NSA and FAV had significant negative correlations with the LCEA. The correlation between the LTV and the two parameters (FAV and IFS) is shown in Figure 4. The FAV was negatively correlated with the LTV. In contrast, the IFS was positively correlated with the LTV.

## Discussion

The key findings of this study revealed that patients with DDH demonstrated significant abnormalities in proximal femoral morphology. First, patients with DDH exhibited significantly increased FAV and decreased LTV compared to BDDH and NH. These external rotational abnormalities of the proximal femur correlated with the severity of DDH, suggesting their importance in hip joint biomechanics. Akiyama et al. similarly reported increased FAV in DDH [8], and Noble et al. noted rotational abnormalities within the femoral diaphysis [14]. Second, the FH-LT was significantly shorter in the DDH group than in BDDH and NH. Argenson et al. previously reported a shortened FH-LT in DDH using radiographic evaluations [9]; however, this study confirmed these findings with more precise 3D CT measurements.

In addition, the αA was significantly lower in the BDDH group compared to NH, indicating that even mild dysplasia contributes to changes in femoral head morphology. Previous studies have noted challenges in measuring the αA accurately in DDH due to the oval shape of the femoral head [10]. Despite this difficulty, our study used CT imaging to measure the αA as precisely as possible. Furthermore, although the CFI showed no significant difference between DDH and NH groups, this finding aligns with Noble et al., who reported that Crowe I DDH femurs were more fluted compared to more severe Crowe types [14].

Another key result was that the FHD in patients with DDH was significantly smaller than that in NH, and the femoral head shape was more elliptical. Wanner et al. hypothesized that femoral head dislocation affects its development, leading to smaller diameters [15]. However, this study excluded patients with a history of hip dislocation, suggesting that a smaller femoral head is not solely a consequence of dislocation but may also be linked to hip instability and insufficient acetabular coverage. Steppacher et al. similarly reported that DDH is associated with a flattened, elliptical femoral head, which exacerbates hip joint incongruence and contributes to early degenerative changes [10].

The findings of this study have significant clinical implications. First, the external rotational abnormalities, including increased FAV and decreased LTV, may lead to the closer proximity of the proximal femur to the ischium, contributing to ischiofemoral impingement (IFI). IFI results from a narrowed space between the lateral aspect of the ischium and the lesser trochanter, causing load-dependent pain in the lower gluteal and medial thigh regions [16-18]. Second, the shortened FH-LT in DDH may stress the iliopsoas muscle due to internal rotation of the iliac wing and external rotation of the lesser trochanter, which can lead to psoas bursitis. Psoas bursitis may cause hip pain, snapping hip syndrome, or flexion contractures [19]. Finally, the flattened and elliptical femoral head in DDH not only results from hip instability but also exacerbates incongruence in the hip joint, leading to progressive joint degeneration [11].

This study has several limitations. First, the data were collected from specific hospitals in Japan, which may limit generalizability to other populations or regions. Further studies with larger, more diverse cohorts are needed to confirm these findings. Second, while 3D CT models provided accurate measurements, their construction requires expertise, and some limitations in interpretation may remain. Third, although this study identified significant morphological abnormalities, the direct correlation between these findings and clinical symptoms or prognosis was not explored. Further research is necessary to clarify the clinical impact of these abnormalities. Finally, while the sample size was sufficient for analysis, a larger cohort may provide further insights into sex-based differences and improve statistical reliability.

Literature review and comparison

The findings of this study complement and extend previous research, including our earlier work on posterior impingement in DDH [20]. In the earlier study, we demonstrated that posterior impingement and restricted range of motion (ROM) during hip flexion and external rotation are common in patients with DDH. However, the mechanisms underlying these functional limitations were not fully understood. The present study fills this gap by providing a detailed analysis of proximal femoral morphology and its role in hip biomechanics.

While the previous study highlighted posterior impingement, it did not investigate the specific morphological factors contributing to this phenomenon. In contrast, our study demonstrates that decreased LTV and increased FAV are critical contributors to the restricted ROM observed in DDH. Through detailed analysis of the proximal femoral morphology, we showed how these structural abnormalities reduce the IFS, exacerbating limitations in hip adduction and external rotation. This, in turn, increases the risk of posterior impingement and contributes to hip instability findings that were not addressed in the earlier study.

Furthermore, our study introduces a novel perspective on how proximal femoral abnormalities affect hip biomechanics in a dynamic context. By linking reduced LTV to functional limitations, we were able to show how these structural features influence joint stability and dislocation risk during motion. This dynamic analysis was absent in the previous work, which primarily focused on clinical observations of posterior impingement without exploring the underlying anatomical mechanisms.

Importantly, our findings challenge the conventional understanding of hip dislocation mechanisms in DDH. While the earlier study associated posterior impingement with reduced ROM during flexion and external rotation, our study emphasizes the role of proximal femoral morphology in modulating these biomechanical outcomes. This new insight not only enhances our understanding of hip instability in DDH but also has implications for improving surgical strategies, such as implant alignment in THA.

In summary, while the previous study identified posterior impingement as a functional limitation in DDH, the present research advances this understanding by linking this condition to specific proximal femoral morphological abnormalities, particularly decreased LTV. By emphasizing the interplay between these structural factors and hip biomechanics, our study provides new insights into the mechanisms of hip instability and informs strategies to optimize surgical outcomes in patients with DDH.

## Conclusions

This study provides novel insights into the 3D proximal femoral morphology in DDH and BDDH, highlighting significant external rotational abnormalities, shortened FH-LT, and elliptical femoral head shapes. These findings correlate with the severity of DDH and suggest a potential role in the development of clinical symptoms, including ischiofemoral impingement, psoas bursitis, and early degenerative joint disease. Future research should focus on understanding the clinical implications of these morphological changes to improve diagnostic criteria, treatment strategies, and outcomes for patients with DDH and BDDH.
